# Mental Health Crisis and Policing Demand

**DOI:** 10.23889/ijpds.v9i5.2505

**Published:** 2024-09-18

**Authors:** Jeff Bakal, Erik Youngson, Scott Garrison

**Affiliations:** 1 Alberta Health Serivces; 2 University of Alberta

The randomized clinical trial framework has proven to be a robust way of evaluating and comparing medications and procedures for many years. The promise of measured and unmeasured balance and equipoise through randomization remains very appealing. Emerging challenges for trialists have been diminishing returns in terms of effect sizes as treatments become more effective and the ever-growing costs associated with the operation of clinical trials.

The costs and operational overhead limit the ability of publicly funded trials to operate at a sufficiently robust level. Recently we have been working to engage clinicians and patients in pragmatic clinical trials which in part leverage routinely collected health system data to both provide an unbiased and effective means of obtaining high quality data for recruiting, randomization and outcomes.

As these trials have expanded between provincial jurisdictions, we have had to look for creative solutions to assembling and sharing data in order to reach the trial goals. This presentation will examine some of the technical and logistical challenges that we have faced, how they have been addressed and the path forward for clinical trials.

